# Novel hydrophobic butyl rubber damping composites modified with bio-based PF/DBA via the construction of a three-dimensional network

**DOI:** 10.1038/s41598-024-55823-x

**Published:** 2024-02-29

**Authors:** Zhenguo Hu, Zeyu Chen, Fuliang Meng, Yimiao Zhang, Yufei Jia, Hongwei Fei, Songjun Li, Xinhua Yuan

**Affiliations:** 1 School of Materials Science and Engineering, Jiangsu University, Zhenjiang, 212013 Jiangsu China; 2 Changzhou Haoda Technology Co., Ltd., Changzhou, 213133 Jiangsu China; 3 Hangmo New Materials Group Co., Ltd., Huzhou, 313310 Zhejiang China

**Keywords:** Damping rubber, Biomass-modified phenolic resin, Lignin, Damping material, Hydrophobicity, Engineering, Materials science

## Abstract

It is of interest to develop wide-temperature domain damped hydrophobic materials. In this paper, we designed incorporating bio-based phenolic resin into the IIR matrix and introducing dibenzyl fork acetone (DBA) into the main chain structure with sodium hydroxide activation to construct three-dimensional network. In this paper, we designed incorporating bio-based phenolic resin into the IIR matrix and introducing dibenzyl fork acetone (DBA) into the main chain structure with sodium hydroxide activation to construct three-dimensional network. The added bio-based phenolic resin has reticulated structure blended with butyl rubber, combined with sodium hydride activation-modified IIR. The results show that sodium hydride activated modification of DBA is introduced into the main chain structure of IIR by infrared and ^1^H NMR analysis. The material hydrophobic is realized by the introduction of DBA with static water contact angle of 103.5°. The addition of 10phr lignin-based phenolic resin (LPF) is compatible with IIR, and the torque can reach 7.0 N-m. The tensile elongation of the modified butyl rubber composite can reach 2400% with tensile strength up to 11.43 MPa, while the damping factor can reach 0.37 even at 70 °C. The thermal stability of the composites is enhanced with mass retention rate of 28%. The bio-based PF/NaH activation-modified butyl rubber damping material has potential applications in damping hydrophobicity with wide temperature range.

## Introduction

With the continuous development of the times and the progress of life, noise pollution and mechanical vibration have become inevitable problems in human life^1^. People’s demand for a living environment is increasing, and mechanical vibration and its generated noise make people unable to tolerate it^2^. Not only that, mechanical vibration makes the accuracy and life of the machine also suffer^3^. Sound-absorbing materials have wide range of application prospects, and sound-absorbing materials have excellent damping properties^4^.

Rubber-like materials have excellent viscoelasticity with high internal friction consumption and excellent damping properties. These rubbers generally have high relative molecular mass and strong interactions between molecular chains, such as ionic bonds, hydrogen bonds and polar groups. These rubbers are used as damping materials in vehicles, rail transportation, aerospace and other fields^5^. However, in actual use, the narrow practical damping temperature domain, insufficient damping effect and poor thermal stability limit the application to some extent^6^. Therefore, it is still essential to develop damping rubber composites with excellent damping, and the ideal damping material should be efficiently damped with relevant properties such as mechanical and thermal stability.

IIR is a polymer made from isobutylene and a small amount of isoprene, which is widely used to manufacture inner tubes, anti-vibration rubber, industrial rubber sheets and other fields due to its distinct properties^7,8^. IIR has large number of methyl groups on the macromolecular chain, and the internal friction between the molecular chains is considerable, while the damping encountered during the relaxation process is converted into internal consumption through the macromolecular chains. IIR has specific damping effect near the glass transition temperature *T*_g_, where the macromolecular chain segments and the molecular chain hysteresis are free to move^9,10^.

Researchers have developed a series of damping materials by different methods. The common modification methods are adding functional reinforcing fillers into the IIR matrix^11^ or blending with other fillers to prepare composite damping materials. Nanofillers have large specific surface area and can thoroughly rub with the rubber matrix. The joint reinforcing fillers include carbon black, talc and carbon nanotubes^12,13^. From the environmental point of view, Tang^14^ added the waste recycled red brick powder into styrene-butadiene rubber (SBR) instead of the traditional filler carbon black and silica to improve the combination of filler and rubber. The production cost is reduced, and the tensile strength and tensile permanent deformation properties are improved. Experimental tests show that utilizing bamboo charcoal (BB) as natural rubber (NR) filler has synergistic effect on the vibration damping properties of the NR matrix^15^.

Blending the resin^16^ with IIR can effectively improve the damping temperature domain of butyl rubber composites. Song^17^ improved the high-temperature damping properties of butyl rubber/phenolic resin composites by introducing different hindered amine molecules. Using IIR as matrix, additional relaxation components can be introduced to prepare desirable damping materials. Xia^18^ controlled the damping properties of IIR by varying the chain length and content of PIB with damping temperature range up to 115.4 °C and peak tan*δ* of 2.01. The introduction of a larger volume and a certain number of side groups to the molecular structure of rubber utilizing polymerization^19,20^ is an important method to improve the damping properties of rubber. However, the modification reaction is more complex and difficult to control precisely. The grafting rate of introducing side groups on the molecular rubber chain is not high, and this method has some limitations in practical applications.

In recent years, due to the limited and unsustainable petrochemical resources and environmental pollution, sustainable development and green environment have become essential principles in the design of damping materials^21,22^. As a typical biomass material, lignin is one of the rare renewable resources among aromatic compounds, and lignin can be used as substitute for phenol to prepare lignin-based phenolic resins (LPF)^23^. The aromatic part together with intramolecular solid/intermolecular interactions (hydrogen bonding, π-electron interactions) endow the lignin molecule with high rigidity, which makes lignin potential reinforced material for polymeric materials^24,25^. Cured IIR with lignin-modified phenolic resin can improve the properties of butyl rubber.

Because butyl rubber contains polar functional groups, it is generally hydrophilic material and often absorbs moisture during use. The thermal stability is poor, resulting in the traditional damping materials usually having short service life. Developing damping material with good moisture resistance and high thermal stability is imperative. Therefore, this experimental study was conducted to improve the damping properties of the composites from the combination of co-blending modification and NaH activation modification of the IIR main chain structure. The bio-based phenolic resin was prepared by replacing a certain amount of phenol with lignin, which was added to the IIR matrix. On this basis, the IIR was activated with NaH and DBA containing benzene ring, and a double bond was introduced into the IIR main chain structure to prepare broad temperature domain damping hydrophobic butyl rubber composites.

## Experimental section

### Materials

Butyl rubber (IRR1751) was obtained from SINOPEC BEIJING YANSHAN COMPANY. Stearic acid, zinc oxide (ZnO), Tetramethylthiuram disulfide (TMTD, AR), 2,2′-Dithiobis (DM, AR), sulfur powder (S, CP), sodium hydride (NaH), Phenol (99.0wt%), formaldehyde solution (37% wt% in H_2_O), Lignin (Alkaline), dibenzyl ketone (DBA) were purchased from Sinopharm Chemical Reagent Co., Ltd. Montmorillonite was purchased from Hebei Hongyao Mineral Processing Co. The chemical materials were used without any purification. The homemade lignin-based phenolic resin was prepared by our subject group.

### Preparation of the composites

The same amount additives were added to each sample with the sum mass of ZnO, stearic acid, TMTD, DM and S of 12.6 g when IIR was used 100 g. The rest amounts of the added reagents are shown in Table 1.

**Table 1:** Formulation design of bio-based PF/NaH activation-modified butyl rubber damping material.

Sample code	IIR	Montmorillonite	LPF	NaH	DBA
IIR	100	–	–	–	–
IIR/M	100	40	–	–	–
IIR/M/LPF-10	100	40	10	–	–
IIR/M/LPF-15	100	40	15		
IIR/M/LPF-20	100	40	20	–	–
IIR-6DBA/M/LPF-10	100	40	10	–	6
IIR-H2DBA/M/LPF-10	100	40	10	0.21	2
IIR-H4DBA/M/LPF-10	100	40	10	0.42	4
IIR-H6DBA/M/LPF-10	100	40	10	0.63	6
IIR-H8DBA/M/LPF-10	100	40	10	0.84	8
IIR-H10DBA/M/LPF-10	100	40	10	1.05	10

Stearic acid acts as a softening and plasticizing agent and facilitates the dispersion of other fillers. Zinc oxide acts as an activator of the rubber. TMTD and DM are rubber accelerators used to increase the rate of vulcanization of the rubber. Sulfur is used in the vulcanization of the rubber to cross-link the linear chains of rubber molecules into a network. Lignin phenolic resins are added to improve IIR related properties such as damping and tensility.

**Figure 1:**
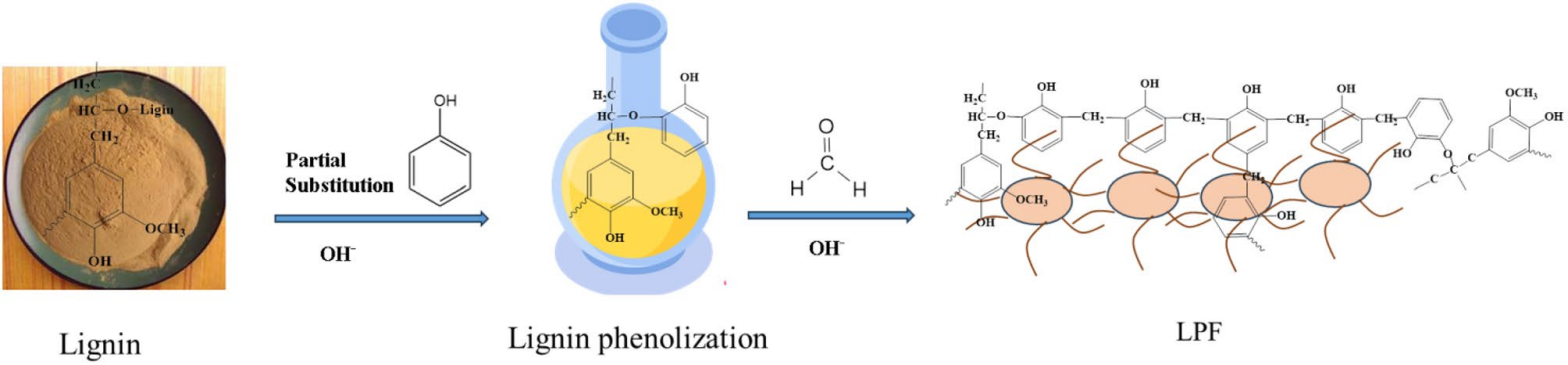
Synthesis of lignin phenolic resin.

Figure 1 shows the flow chart for the synthesis of bio-based phenolic resins. In 250 mL three-necked flask equipped with stirrer, thermometer and reflux condenser, a certain proportion of phenol, NaOH and alkali lignin were respectively added and stirred well, and the temperature of the reaction system was raised to 90 °C and reacted for 90 min to obtain lignin phenol. The lignin mass accounted for 40% of the total mass of lignin and phenol, and the NaOH mass was 6% of the phenol mass. The reaction conditions of the lignin phenolic resin synthesis stage were consistent with the phenolic resin synthesis. When the temperature of the reaction system was reduced to 60 ℃, the formaldehyde was added, stirred uniformly and reacted for 60 min. After that, the temperature of the reaction system was increased to 90 ℃, and the reaction was carried out for 120 min with the molar mass ratio of phenol to formaldehyde of 1: 1.7. The reaction product was cooled to room temperature and then washed three times with respective anhydrous ethanol and deionized water in ultrasonic cleaner, and the product was finally put into vacuum oven and dried at 60 ℃ for 24 h. The product was dehydrated to obtain lignin phenolic resin (LPF).

Lignin was first phenolized by adding phenol under alkaline solution conditions, and formaldehyde was added under alkaline conditions after lignin was phenolized. Finally, the prepared bio-based phenolic resin was dried, crushed and kept for next preparation.

**Figure 2:**
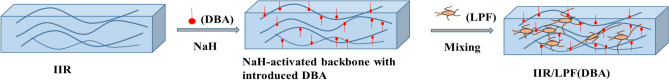
Schematic diagram of the composite structure.

**Figure 3:**
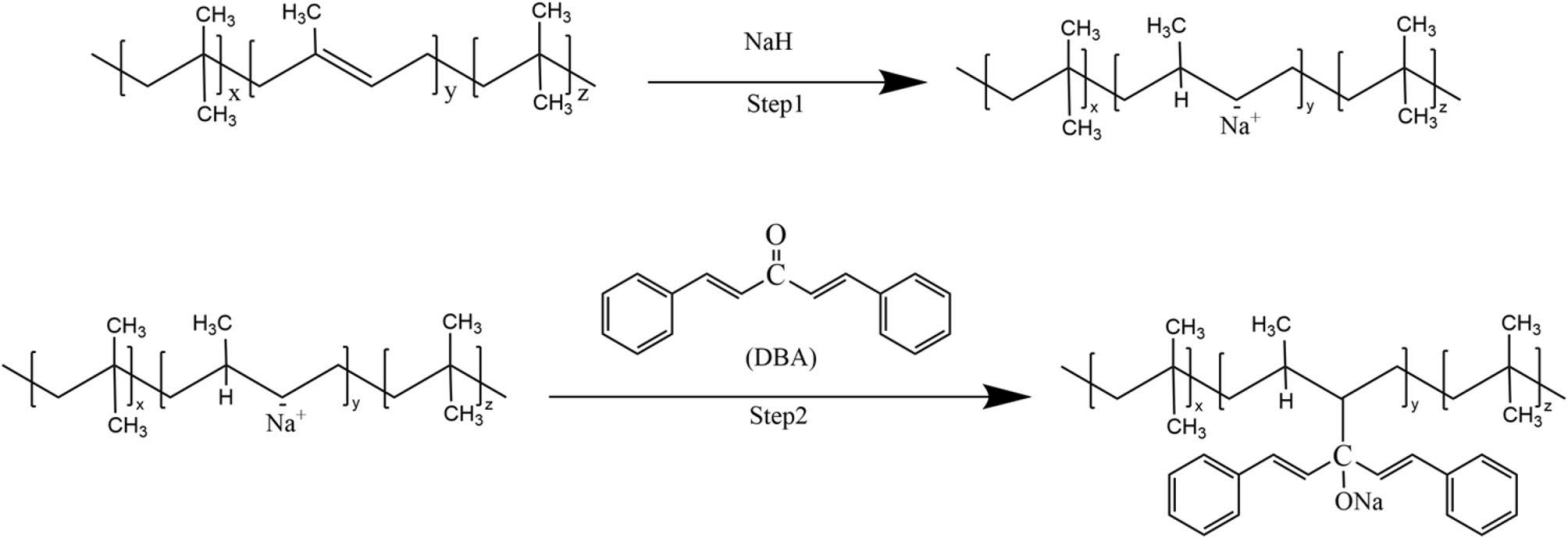
NaH activation modified IIR with introduced DBA.

Figure 2 shows the flow chart for the preparation of damping composites. At a specific temperature, IIR and NaH were added into 60 mL Hacker’s torque rheometer, and after the torque time curve was stabilized, dibenzylidene fork acetone was added to continue the reaction for about 15 min (Fig. 3), and the modified rubber was obtained and set aside.

The rubber added with dibenzyl fork acetone was plasticized on a double-roller opener, and the fillers stearic acid, zinc oxide, TMTD, DM, sulfur, lignin-based phenolic resin and montmorillonite were added in order and blended well. Stearic acid acted as softening and plasticizing agent and facilitated the dispersion of other fillers. Zinc oxide acted as activator of the rubber. TMTD and DM were rubber accelerators to increase the rate of vulcanization of the rubber. Sulfur was used in the rubber vulcanization to cross-link the linear chains of rubber molecules into network. Lignin phenolic resins were added to improve IIR related properties such as damping and ductility. The blended sample was left for 24 h to remove the air bubbles, and the raw rubber was prepared. Finally, 7–10 g of raw rubber was weighed and vulcanized on the plate vulcanizer to prepare the specimens of butyl rubber comprehensive temperature range damping composites. The vulcanization conditions were determined with vulcanization temperature of 160–170 ℃, vulcanization pressure of 10–12 MPa and vulcanization time of 14–18 min.

The isoprene monomer provides the molecular backbone of butyl rubber with an active point where the cross-linking reactions can take place. The most significant advantage of using reactive processing technology of rubber is that the chemical modification reaction can be carried out in general-purpose rubber processing equipment (e.g., torque rheometers, compactors). Short reaction process, simple preparation process and low experimental equipment requirements characterize this solvent-free ontology modification method. According to the principles of organic chemistry, sodium hydride does not react with alkanes. However, under certain conditions, it can react with hydrogen in the olefin double bond or the allyl position to produce carbon-negative ion. The typical reaction of carbon-negative ions is nucleophilic addition to compounds containing carbonyl groups^26^. In this experiment, the NaH activator was selected to produce negative carbon ions on the molecular rubber chain. Then DBA, a compound containing carbonyl groups, was selected to introduce unsaturated double-bond functional groups and benzene rings with larger side groups to the main macromolecular chain. In the butyl rubber modification reaction process, the isobutylene structural unit is assumed not to participate in the reaction because the side methyl group on the isobutylene unit has low reactivity and does not participate in the reaction.

### Characterization

The different functional groups were examined by Fourier transform infrared spectroscopy (Nicolet AVATAR360, Madison). The prepared samples were cut into thin slices and the reflectance spectra of the samples were tested in the mid-infrared band using the attenuated total reflection mode (ATR). The structure of LPF/NaH modified butyl rubber was observed by scanning and analyzing in the wavelength range of 4000–500/cm.

Determination of the vulcanization characteristic curve of rubber mixing was carried out in GT-M3000AU (GOTECH Testing Machines Inc) type rotorless vulcanizer. After the rubber mix was parked for 24 h, about 5 g of rubber mix was cut and placed between the polycool films for measurement.

The Bruker Ultra Shield 400 plus NMR instrument was used to obtain nuclear magnetic resonance (NMR) hydrogen spectroscopy with deuterated chloroform (CDCL_3_) as solvent and tetramethylsilane (TMS) as internal standard at room temperature. Small amount of LPF/NaH modified butyl rubber was dissolved in cyclohexane. When the rubber material was completely dissolved, and it was then precipitated with anhydrous ethanol. The dissolution process was repeated for 2–3 times to dissolve the LPF and inorganic fillers out of the rubber material. Finally, the purified NaH modified IIR was obtained and dried in vacuum drying oven for 48 h, after which they could be subjected to NMR characterization.

Micromorphological analysis was performed by scanning electron microscope (SEM, NovaNano450/FEI). A small portion of the fracture was cut off, the section was gold sprayed, and then the micromorphology at the section was observed by the scanning electron microscope.

To conduct the tensile test, the vulcanized sample was cut into dumbbell type pattern with thickness of 1 mm, width of 2 mm and marking distance of 10 mm. The tensile test was carried out on electronic universal testing machine (SHIMADZU AGS-X) at 100 mm/min, and the load-stroke curve was recorded. Each batch of samples was tested three times.

The dynamic thermo-mechanical analyzer was used to perform temperature scanning in tensile mode with frequency of 10 Hz, amplitude of 5 μm, ramp rate of 3 k/min, starting temperature of − 40 °C and termination temperature of 80 °C. The prepared samples with thickness of 1 mm, width of 2 mm and length of 20 mm were placed on the dynamic thermo-mechanical analyzer for the DMA analysis test, and the curves of tan*δ*, E′, E′′ versus temperature were recorded. The testing procedure followed the standard method. Each batch of samples was tested three times.

The water contact angle of material surfaces was measured by contact angle meter (Dataphysics, OCA15EC-TBU100). After vulcanization, the sample was cut with the size greater than 1 cm*1 cm and fixed on the workbench. The level was dropped water so that the sample surface and water droplets were gently contacted each other, and the image was gained by the software to obtain the contact angle. Samples from each batch were tested three times.

To conduct thermal test, the vulcanized rubber was tested by thermal weight loss analyzer (NETZSCH STA 449 F5) at 10 °C/min rate in nitrogen atmosphere from room temperature to 800 °C.

## Results and discussion

### Infrared spectroscopy analysis

**Figure 4:**
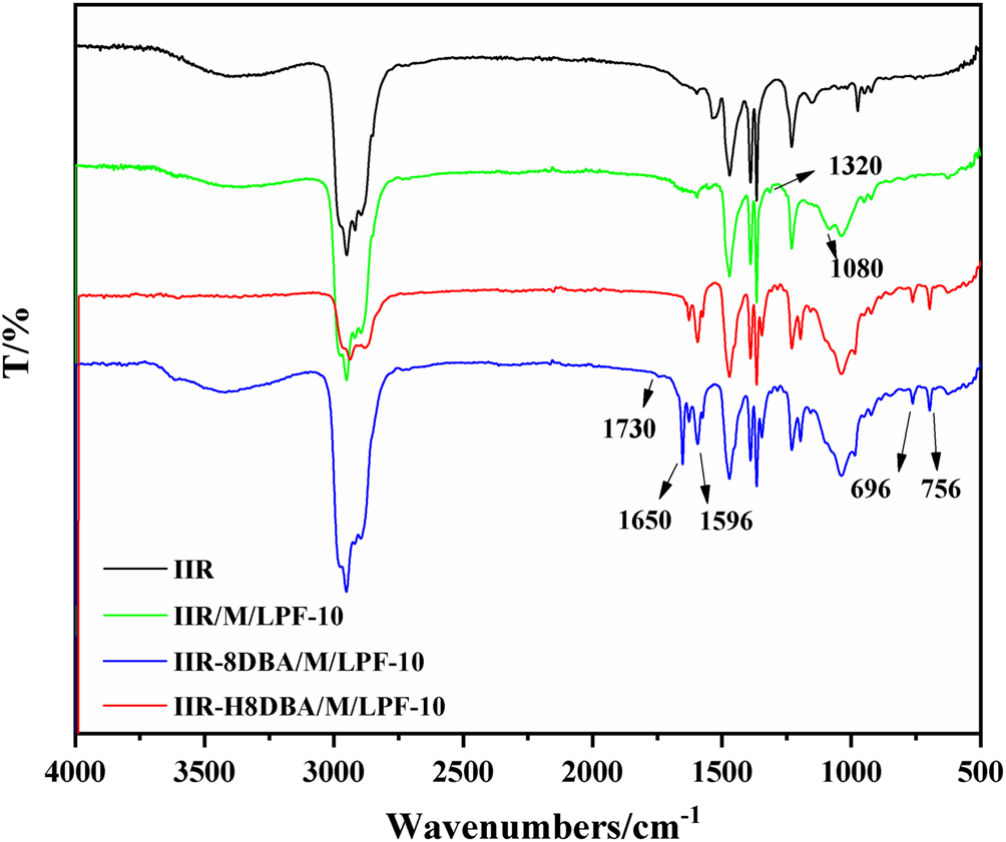
Infrared spectra of IIR and modified IIR composites.

The FT-IR spectra of IIR and modified IIR composites are shown in Fig. 4, and comparative analysis reveals that the composite spectra are different from the IIR spectra. In the IIR/M/LPF curve of Fig. 4, 1320/cm is the C–H stretching vibration of methoxy in lignin, and 1080/cm is the characteristic absorption peak of lignin, which corresponds to the C–O–C stretching vibration of the ether bond of methoxy in lignin^23,27^. The comparison of FT-IR spectra of IIR and IIR/M/LPF reveals that the incorporation of lignin-based phenolic resins and inorganic fillers does not alter the primary structure of the butyl rubber. The IIR-8DBA/M/LPF and IIR-H8DBA/M/LPF curves at 756/cm, 696/cm and 1596/cm are for the benzene ring =C–H stretching bend peaks in DBA^28^. IIR-8DBA/M/LPF curve is for the specimen with DBA but without NaH catalyst, while IIR-H8DBA/M/LP curve is for the specimen with NaH catalyst and DBA. The disappearance of C=O of the carbonyl group at 1730/cm^29^ and 1650/cm in the IIR-H8DBA/M/LPF curves indicates the incorporation of DBA into the main chain structure of butyl rubber. According to the above analysis, the added LPF and inorganic filler does not change the IIR main chain structure, and the use of NaH can activate the IIR double bond structure to introduce DBA into the IIR main chain structure.

### Curing performance analysis

Rubber vulcanization were conducted obtained by rubber vulcanizer to obtain the amount of torque during the vulcanization process of rubber. According to the torque cure time curve, it can be used to characterize the vulcanization properties of rubber. In the process of vulcanization, there is a certain proportional relationship between the torque of the rubber and the cross-linking density, so the vulcanization characteristic curve can be used to characterize the rubber in the vulcanization process to form a cross-linking network.

**Figure 5:**
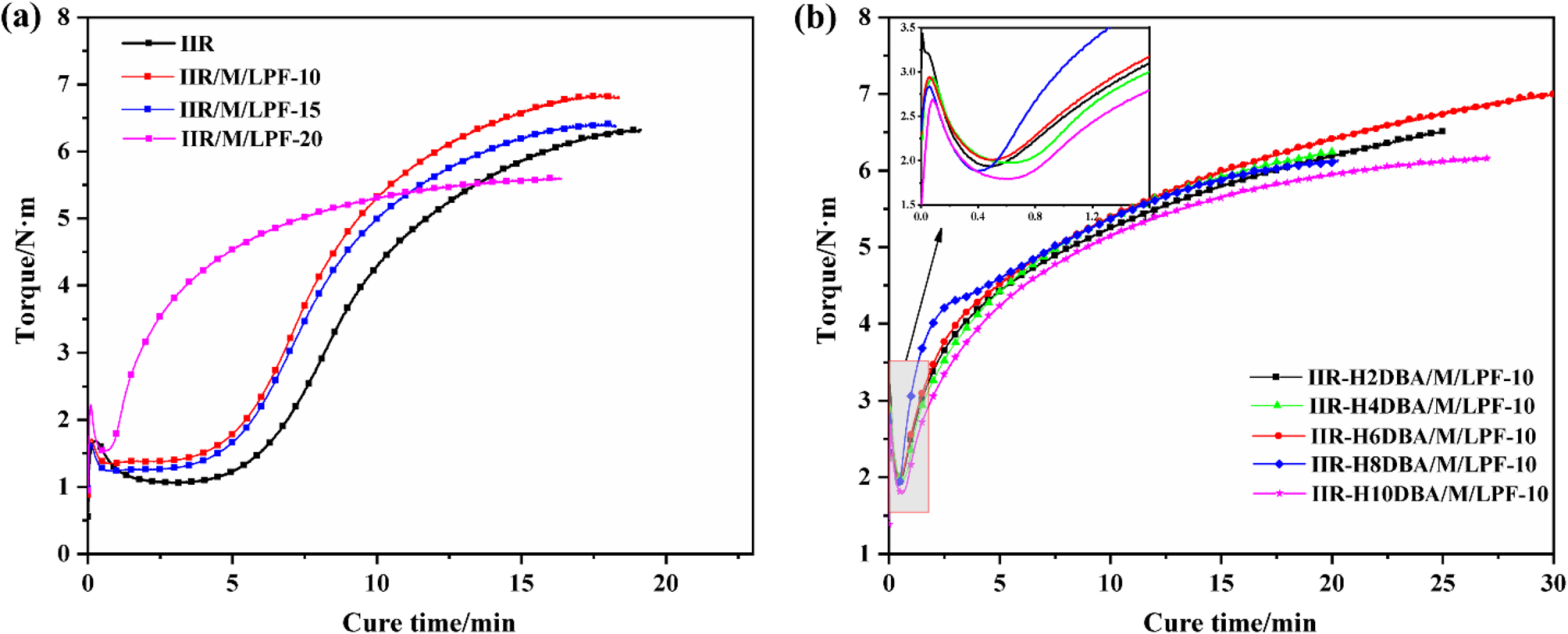
Characteristic vulcanization curves for IIR and modified IIR composites.

Figure 5a shows the vulcanization characteristic curves of IIR, IIR/M/LPF at 160 °C. IIR vulcanization first undergoes induction period, the vulcanization curve is relatively smooth, and there is no formation of cross-linking network in the induction period. As the vulcanization curve rises, IIR molecules form cross-linking network, making them somewhat elastic, and at this stage, the vulcanization curve rises rapidly. Finally, there is a curing stage where the inter-molecular cross-linking network is basically formed, and the torque is finally kept constant with final torque of 6.3 N m. From Fig. 5a, it can be found that the vulcanization induction period of IIR/M/LPF is shortened, and the final vulcanization torque is increased. Lignin has a certain network structure, and the prepared LPF and IIR interpenetrate each other and form a certain three-dimensional network structure after vulcanization^30^. The torque is increased during vulcanization. Compared with IIR vulcanization curve, the addition of 10 phr LPF can achieve 6.8 N m of torque. When adding 20 phr LPF, the final vulcanization torque is slightly smaller than that of IIR. This is because the mixing compatibility between LPF and IIR becomes poor due to the addition of too much LPF.

Figure 5b shows the vulcanization characteristic curves of IIR composites with the addition of NaH and DBA. From Fig. 5b, it can be found that the induction period is significantly shortened in the vulcanization curve after adding DBA. The IIR vulcanization curves with the addition of DBA show sharp increase in the torque of composite at the early stage of vulcanization. This is due to the introduction of DBA into the IIR main chain structure using NaH, which changes the original structure of IIR incorporation. As the amount of DBA introduction is increased, the torque of vulcanization is first increased and then decreased, and the final torque of vulcanization reaches 7.0 N m when 6 phr of DBA is added.

### ^1^H NMR analysis

**Figure 6:**
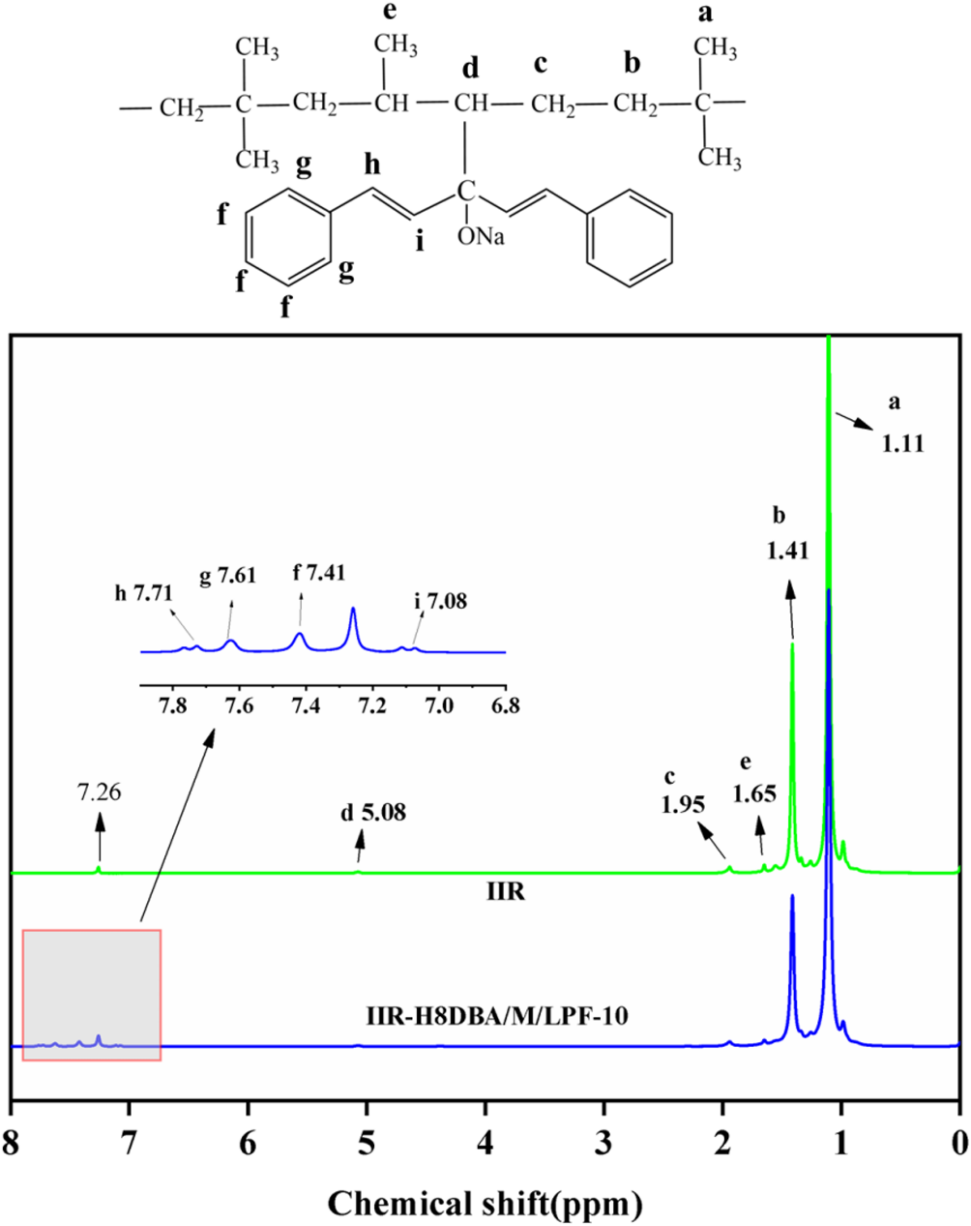
^1^H-NMR spectra of IIR and IIR modified with NaH.

The structures of NaH/DBA modified IIR and pure IIR were characterized by ^1^H-NMR to provide a basis for the introduction of DBA into IIR by NaH-activated IIR. ^1^H-NMR spectra are shown in Fig. 6. In the IIR NMR spectrum, two strong and one weak proton signals are detected, which are attributed to –CH_3_ (δ 1.11 ppm, 3 H) and CH_2_– (δ 1.41 ppm, 2H) in isobutene^31^ and –C(CH_3_)=CH– (δ 5.08 ppm, 1H) group in isoprene^32,33^. The new shifts appear in the NMR spectra of NaH activation-modified IIR, corresponding to –HC=CH– (δ 7.08 ppm) and –CH=CH– (δ 7.71 ppm) in the DBA, respectively. The characteristic peaks at 7.41 ppm and 7.61 ppm, corresponding to the two types of H on the benzene ring in DBA^34^, are shown in Fig. 6. The auxiliary analysis in the infrared spectrum of Fig. 4 was performed by comparing the addition of activator and the absence of activator. After adding the NaH activator, the C=O of the carbonyl group disappeared, indicating that DBA was introduced into the main chain structure of the large IIR. Combined with ^1^H-NMR spectra and IR analysis, it can be shown that DBA can be introduced into the IIR main chain structure via the NaH activator.

### SEM morphology analysis of composite cross-section

**Figure 7:**
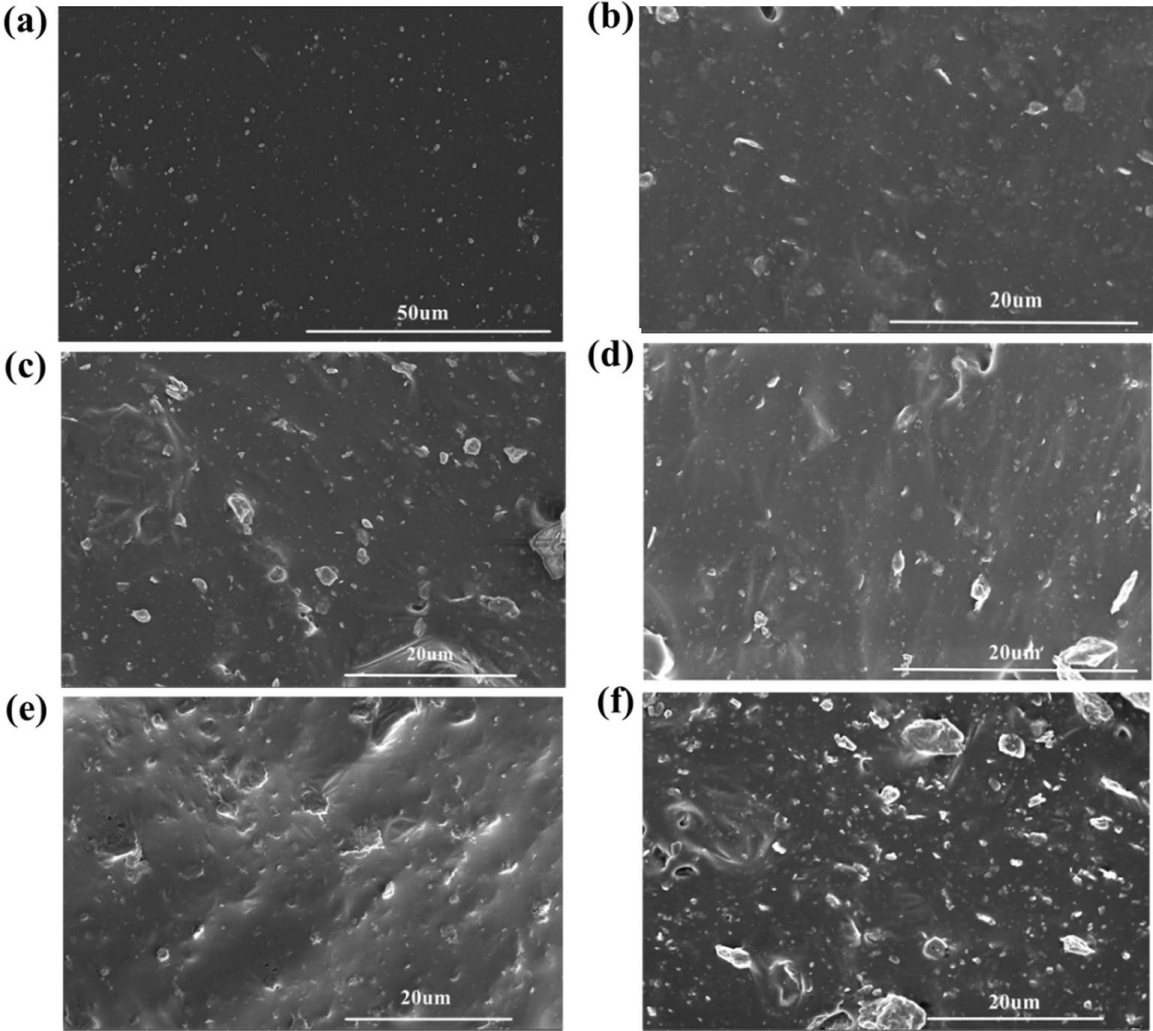
SEM structural photos of the section ((**a**) IIR, (**b**) IIR/M/LPF-10), (**c**) IIR/M/LPF-20, (**d**) IIR-H2DBA/M/LPF-10, (**e**) IIR-H6DBA/M/LPF-10, (**f**) IIR-H10DBA/M/LPF-10).

The cross-sectional morphology of IIR and bio-based PF/NaH activation-modified butyl rubber damping composites are shown in Fig. 7. The morphology and structure of polymer blending systems are complex and varied, and changes in the size of the components of each design can cause great changes in the physical properties of the composites, so it is necessary to study the morphology and structure of the composites. The morphological structure of composites can generally be observed and analyzed by scanning electron microscopy. Figure 7a shows the IIR tensile section morphology. From the electron micro-graphs, it is found that the section is relatively smooth, which is due to the addition of only some necessary vulcanization additives. From the cross-sectional morphology of IIR/M/LPF-10 and IIR/M/LPF-20 in Fig. 7b and c, it can be seen that the addition of inorganic fillers montmorillonite and LPF increases the roughness of the cross-sectional morphology of the composites. The added filler particles are in close contact with the substrate and are well dispersed and compatible. Figure 7d–f show the cross-sectional morphology of IIR-H2DBA/M/LPF-10, IIR-H6DBA/M/LPF-10 and IIR-H10DBA/M/LPF-10. When the amount of DBA added is within 6 phr, the introduction of DBA into the IIR backbone does not affect the cross-sectional structure of the composite. With the addition of excessive amounts of NaH and DBA, it is found from the scanned cross-section morphology that DBA agglomerates in the IIR matrix and can affect the properties of butyl rubber damping materials.

### Tensile property analysis

**Figure 8:**
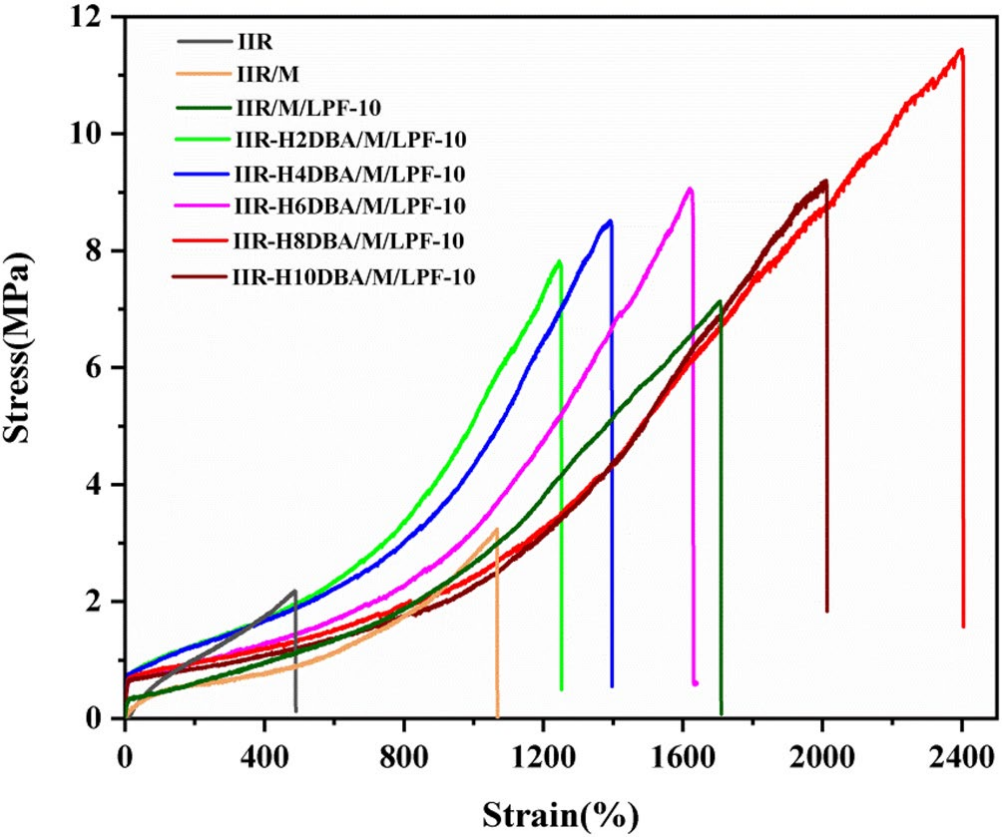
Stress–strain curves of IIR and bio-based PF/NaH activation-modified butyl rubber damping materials.

Figure 8 shows the stress–strain curves of IIR with bio-based PF/NaH activation-modified butyl rubber. It can be seen in Fig. 8 that the tensile properties of the damping composites prepared by adding bio-based PF in combination with NaH activation-modified butyl rubber are significantly improved compared to that of pure IIR. The experimental results show that the composites' tensile properties become better because the added fillers and LPF have more significant effect on the tensile of the composites. Lignin itself has a specific network structure, and the addition of LPF and IIR matrix forms a specific interpenetrating network structure. Adding LPF improves the fracture strength of the composite and delays the occurrence of its fracture, and the addition of LPF powder particles and the interface of the IIR interface interacts with the deformation of the interfacial slip. The introduced dibenzyl fork acetone (DBA) forms certain hydrogen bonds with LPF, and the molecular chains are cross-linked and entangled with each other to enhance the tensile properties to a certain extent.

The specific tensile properties are shown in Table 2. From the comparison of the tensile properties in Table 2, it is found that the combination of bio-based PF and NaH activation modification can increase the elongation at break and tensile strength of IIR damping materials, and the hardness of IIR composites is also changed. The elongation at break of IIR-H8DBA/M/LPF reaches as high as 2400% with the breaking strength as high as 11.4 MPa in the prepared samples. Inorganic fillers and bio-based phenolic resins have some effect on the hardness of damping composites. The introduction of DBA has little effect on the hardness of butyl rubber damping materials. The added inorganic filler and lignin-based PF can increase the stiffness of the prepared composites because LPF is thermosetting resin with high strength and modulus, which has reinforcing effect on the rubber.

**Table 2:** Tensile properties of IIR and bio-based PF/NaH activation-modified butyl rubber damping materials.

Sample	Stress at break/MPa	Strain at break/%	100% tensile stress/MPa	300% tensile stress/MPa	Shore A hardness/°
IIR	2.17	488	0.63	1.35	37
IIR/M	3.19	1059	0.45	0.66	43
IIR/M/LPF-10	7.13	1706	0.44	0.78	46
IIR-H2DBA/M/LPF-10	7.77	1242	1.03	1.45	46
IIR-H4DBA/M/LPF-10	8.40	1345	1.01	1.44	47
IIR-H6DBA/M/LPF-10	9.00	1612	0.82	1.11	47
IIR-H8DBA/M/LPF-10	11.43	2400	0.83	1.05	49
IIR-H10DBA/M/LPF-10	9.00	2010	0.75	0.95	50

### Dynamic mechanical properties testing

The damping factor and molecular dynamics changes of butyl rubber and bio-based PF/NaH activation-modified butyl rubber damping composites were examined using dynamic thermomechanical analyzer.

**Figure 9:**
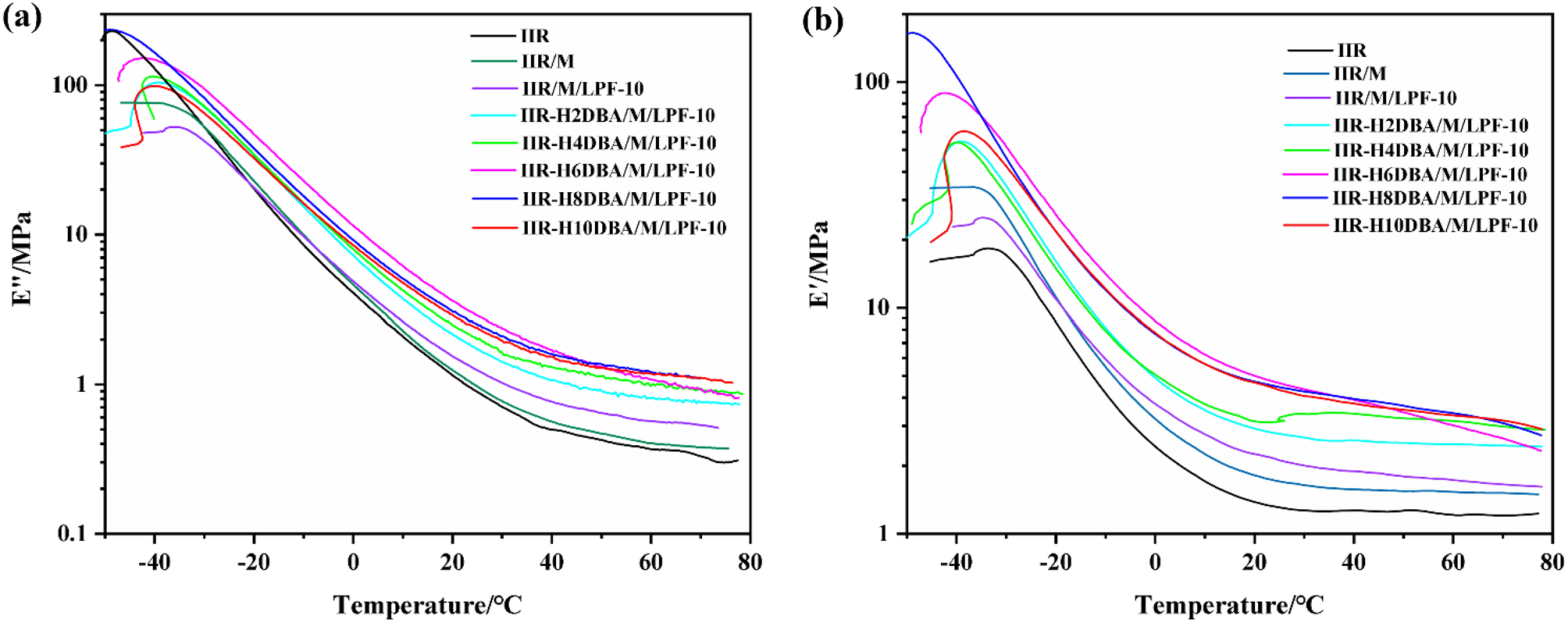
IIR and different ratios of NaH activation-modified butyl rubber composites (a) loss modulus, (b) energy storage modulus.

The temperature dependence of loss modulus and energy storage modulus of IIR and bio-based PF/NaH activation-modified butyl rubber damping composites are shown in Fig. 9. The peak modulus of the biobased PF/NaH-modified IIR composites is lower than that of IIR at the glass transition temperature (*T*_g_), and the modulus of IIR is higher at lower temperatures. From the loss modulus (*E′′*) curve in Fig. 9a, it can be found that the loss modulus *E′′* of the composite is increased, which is higher than that of the pure IIR. This is because the molecular rubber chain starts to move after *T*_g_, and the hydrogen bonds formed between the added bio-based PF molecules and between the added bio-based PF and the DBA introduced on the IIR main chain, consuming some energy and increasing the loss modulus. The energy storage modulus (*E′*) is increased with the introduction of DBA in Fig. 9b because intermolecular interactions can constrain the mobility of the polymer chains. The bio-based PF 3D network structure, the incorporated inorganic filler montmorillonite and the introduced DBA can enhance the stiffness of the composites, thus increasing the storage modulus of the composites.

**Figure 10:**
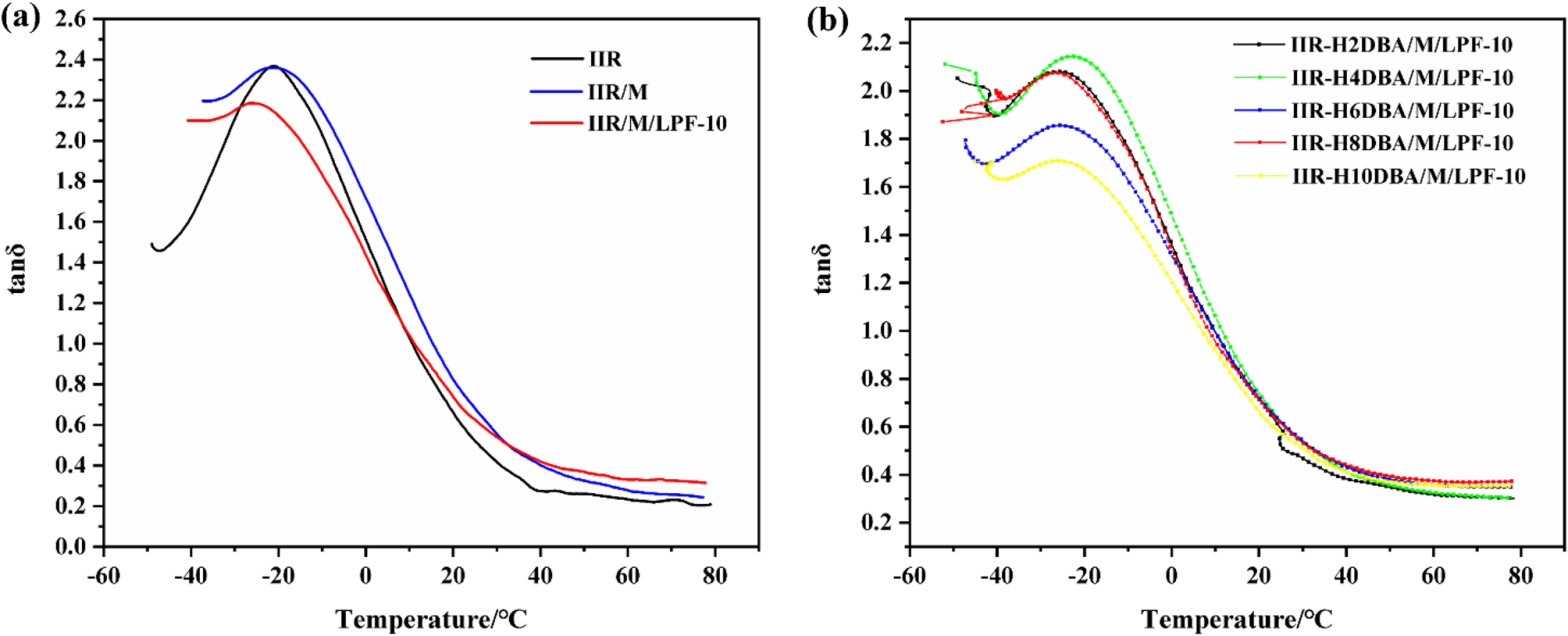
Damping factor of NaH activation-modified butyl rubber composites with different ratios.

Figure 10a and b show the variation of the damping factor with temperature for IIR and bio-based PF/NaH activation-modified butyl rubber damping composites. The damping performance of the composite material is significantly improved compared with that of IIR. The tan*δ* curves of the modified butyl rubber damping material have only single peak as the same as pure IIR tan*δ* curve. Obvious reduction can be seen in tanδ peak height of butyl rubber composites modified by adding bio-based PF and NaH. This indicates good compatibility between bio-based PF and DBA introduced by NaH modification and IIR. However, the tan*δ* peak of the damped composites is smaller than that of IIR due to the enhanced interaction between the incorporated bio-based PF 3D mesh and the introduced DBA and IIR chains, which constrains the migration rate of the polymer chains.

Figure 10a shows that the pure IIR has almost no damping effect at room temperature and above. The damping temperature range of bio-based PF/NaH activation-modified butyl rubber damping composites with tan*δ* > 0.3 is broadened and still has damping effect at room temperature and above. Figure 10b shows the effect of DBA content on the damping factor at different temperatures. The damping factor of the prepared IIR-H8DBA/M/LPF-10 can reach 0.37 even at temperature of 70 °C. When the temperature is higher than *T*_g_, the modified butyl rubber composite is subjected to alternating stress, and the molecular rubber chains and molecular segments start to move. Lignin has a certain network structure, and a three-dimensional network structure is formed between the added LPF and IIR, and physical friction is generated between the LPF and the IIR matrix, during which kinetic energy is converted into heat and part of the energy is consumed. Since the bio-based PF has certain polar groups, many hydrogen bonds are formed between LPF molecules and between LPF and the introduced DBA, and the hydrogen bonds are alternately broken and rebuilt under the action of external forces, which consume part of energy. Breakage and regeneration of hydrogen bonds give the composite continuous damping effect^35,36^. When more DBA is added, the excess activator NaH and DBA are equivalent to fillers, which cannot provide polar groups for the molecular chain of butyl rubber and the aggregation phenomenon within the polymer is serious, the molecular chain movement is limited, and the damping factor shows a decreasing trend.

### Water contact angle analysis

**Figure 11:**
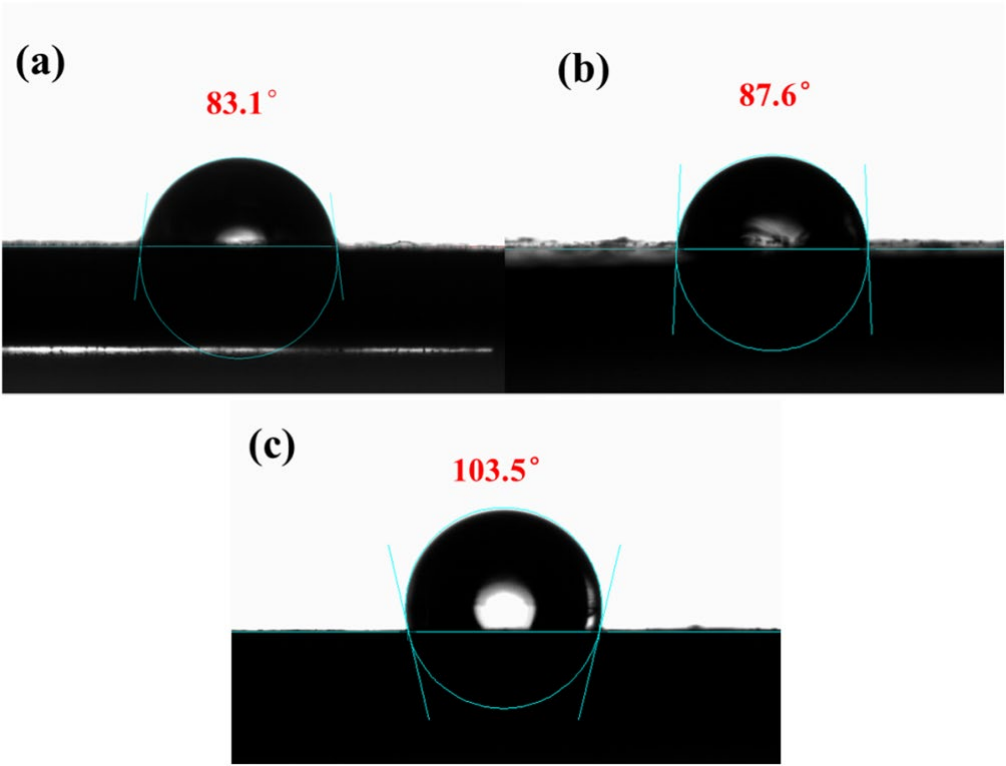
Water contact angle ((**a**) IIR, (**b**) IIR/M/LPF-10, (**c**) IIR-H8DBA/M/LPF-10).

Figure 11 shows the water contact angle of IIR and modified butyl rubber composites. Since butyl rubber contains polar functional groups and is hydrophilic material, the moisture absorption phenomenon often occurs during the use process, coupled with poor thermal stability, resulting in the service life of traditional damping materials being short. It is imperative to develop a damping material with good moisture resistance and high thermal stability. The common method for improving hydrophobic properties is to incorporate inorganic particles into the matrix or combine them with coating to form micro-scale structure and then fill the surface and the micro-structure with nanostructured hydrophobic materials in between^37^. The material is considered hydrophobic when the water contact angle is more than 90°. The water contact angle of IIR and NaH activation-modified butyl rubber composites were tested and analyzed by a water contact angle meter and shown in Fig. 11. The water contact angle test show that IIR exhibits hydrophilic performance with water contact angle of 83.1°.

**Figure 12:**
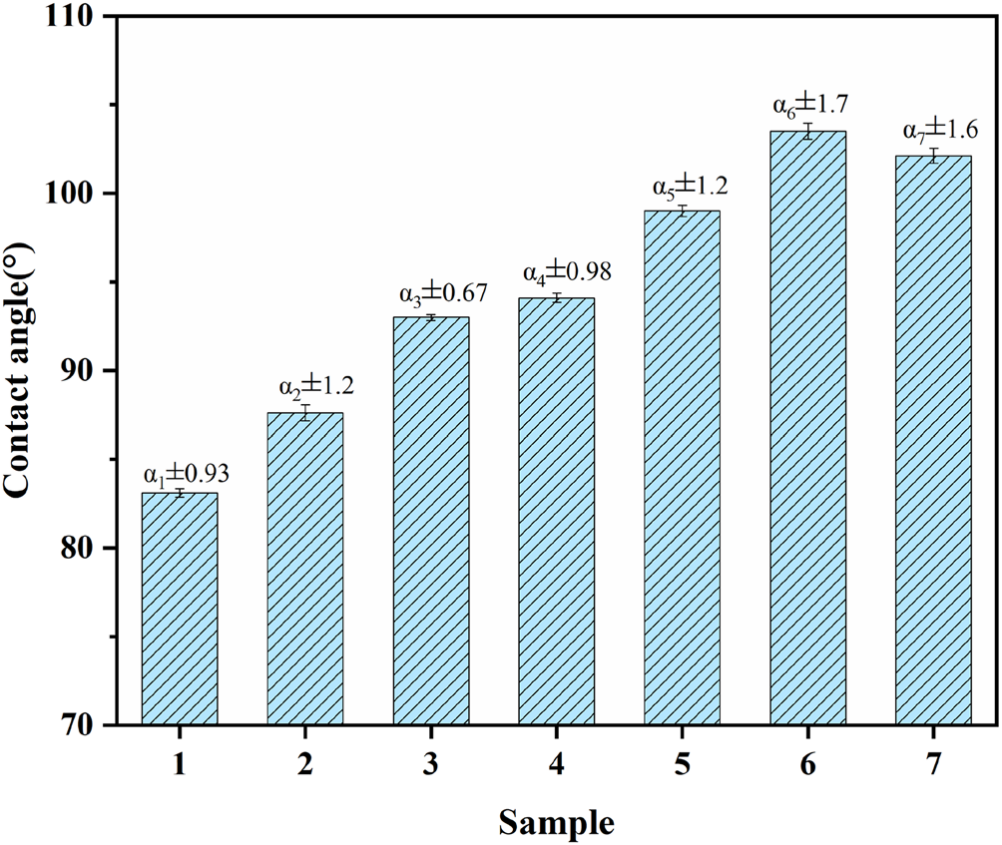
Water contact angle of composites with different content of DBA (1, IIR; 2, IIR/M/LPF-10; 3, IIR-H2DBA/M/LPF-10; 4, IIR-H4DBA/M/LPF-10; 5, IIR-H6DBA/M/LPF-10; 6, IIR-H8DBA/M/LPF-10; 7, IIR-H10DBA/M/LPF-10).

Figure 12 shows the water contact angle of composites with different addition rates of DBA. Preparation of hydrophobic materials can be done by incorporating inorganic particles that synergize with organic components to improve the hydrophobic properties of the material^38,39^. After adding lignin-based phenolic resin and NaH activation-modified, it can be seen that the water contact angle is increased, and the static water contact angle can reach 103.5°, which illuminates that the damping material changes from non-hydrophobic to hydrophobic state. Although the added LPF has certain hydroxyl groups, the NaH activation-modified butyl rubber composite contains double bonds and large number of benzene rings grafting into the main chain structure of butyl rubber. The introduction of large number of aromatic groups in the IIR backbone improves the hydrophobic properties of butyl rubber-damping composites. Adding a certain amount of inorganic filler montmorillonite changes the roughness of the rubber surface. Based on the measured experimental data, the standard deviation α of the samples was calculated at 95% confidence intervals and is indicated in Fig. 12. It can be seen from Fig. 12 that the water contact angle tends to increase first and then decrease after the introduction of DBA in IIR. This is because after the addition of excess DBA, the excess DBA cannot be introduced into the IIR main chain structure, and NaH and DBA are added to the IIR only as fillers, affecting the structure of the composite.

### Thermal degradation characteristics

The vulcanized rubber was tested in a heat loss analyzer model STA499F3 from NETZSCH, Germany, at a rate of 10 °C/min in nitrogen atmosphere from room temperature to 800 °C.

**Figure 13:**
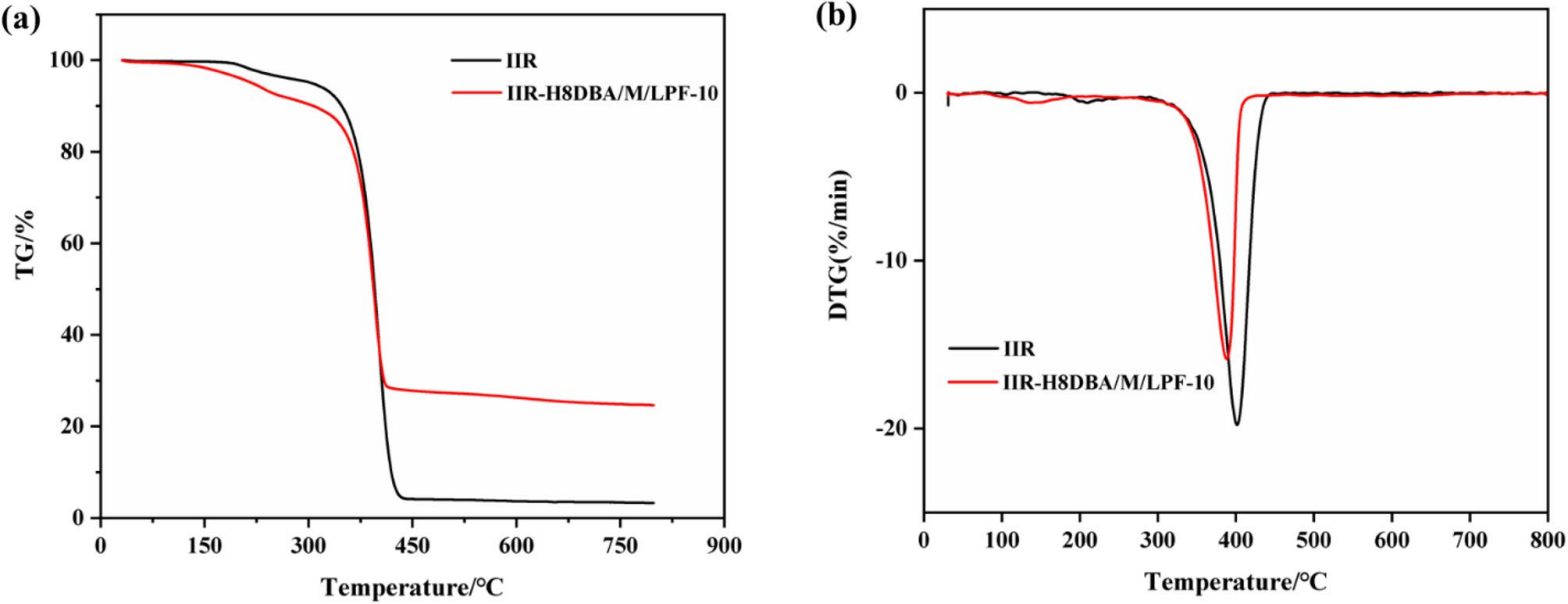
IIR and IIR-H8DBA/M/LPF-10 ((**a**) TG, (**b**) DTG).

Thermogravimetric (TG) analysis can observe the thermal stability of the material. After curing, phenolic resins have excellent thermal stability, relying on their aromatic structure and crosslinking density^40^. The lignin-based phenolic resin (LPF) was prepared, and the thermal stability of the lignin-based PF/NaH activation-modified butyl rubber damping material was compared with that of the unmodified IIR to compare the difference in thermal weight loss characteristics. Figure 13 shows the thermal degradation characteristics of IIR and bio-based PF/NaH activation-modified butyl rubber damping materials tested in this experiment under nitrogen atmosphere conditions, where the studied samples undergo non-oxidative decomposition. The mass loss in the first stage of IIR is the reduction of water molecules and some small molecules inside the polymer. This process takes place at the temperature from 220 °C up to about 375 °C. The mass loss of IIR-H8DBA/M/LPF-10 in the first stage is due to the decomposition of some water molecules in the composite as well as lignin in LPF at high temperatures (over 200 °C). In the second stage of thermal decomposition, the IIR double bond structure and the lateral methyl group are decomposed, and both IIR and IIR-H8DBA/M/LPF-10 start to decompose faster at 350–450 °C. The TG curve of IIR-H8DBA/M/LPF-10 includes the decomposition of dibenzyl acetone. However, the mass retention of the modified butyl rubber damping composites with the addition of bio-based PF/NaH activation is 28% at temperatures above 750 °C. In contrast, the mass retention of pure IIR is only 3.5%. This may be due to the complex structure of lignin and the higher residual carbon rate of LPF, which increases the residual carbon rate of the damping composites. The experimental results show that the mass retention of the damped composites can be improved by adding bio-based LPF and dibenzyl fork acetone, which improvs the composites' thermal stability.

## Conclusions

In this study, the prepared LPF was incorporated into the IIR matrix and combined with NaH activation modification to construct three-dimensional network. Adding inorganic fillers and LPF does not change the IIR backbone structure, and NMR spectral analysis illuminates that DBA is introduced into the IIR backbone structure. IIR have respective breaking stress and breaking elongation of 2.3 MPa and 401%, while LPF/IIR (8DBA) have the improved breaking stress and breaking elongation with breaking stress of up to 11.43 MPa and breaking elongation of up to 2400%. IIR has no damping effect (tanδ < 0.3) above room temperature, while the prepared IIR-H6DBA/M/LPF-10 have damping factor as high as 0.37 even at the temperature of 70 °C. The water contact angle of IIR is 83.1°, which has no hydrophobic effect. When DBA is introduced into the main chain structure of IIR up to 8 phr, the water contact angle can reach 103.5°. The thermal stability of IIR is low, and the mass retention at 800 °C is only 3.5%. The thermal stability of the damping composite is improved with the mass retention of 28% at 800 °C for IIR/H8DBA/M/LPF-10. It is worth noting that the LPF added in this experiment, lignin itself has specific network structure, lignin instead of part of the phenol synthesized LPF, added to the IIR and IIR interpenetration to form a three-dimensional network structure. The addition of LPF and IIR at the same time for sulfurization can improve the crosslinking density of the material. Moreover, the alternative use of lignin can reduce the amount of phenol and residual formaldehyde in the product, saving resources and reducing costs. In conclusion, the new hydrophobic butyl rubber damping composites modified with bio-based PF/DBA were prepared with better hydrophobicity, tensile properties, thermal stability and improved damping factor to widen the damping temperature range, which has better application prospect.

### Supplementary Information

Supplementary Information.

## Data Availability

All data generated or analysed during this study are included in this published article and its supplementary information files.
